# Clinical Utility of ^18^F-2-Fluoro-deoxy-d-glucose PET Imaging in Locally Advanced Esophageal/Gastroesophageal Junction Adenocarcinoma

**DOI:** 10.3390/diagnostics13111884

**Published:** 2023-05-28

**Authors:** Darren Cowzer, Fergus Keane, Geoffrey Y. Ku

**Affiliations:** 1 Department of Medicine, Memorial Sloan Kettering Cancer Center, New York, NY 10065, USA; cowzerd@mskcc.org (D.C.); keanef@mskcc.org (F.K.); 2 Department of Medicine, Weill Cornell University, New York, NY 10065, USA

**Keywords:** esophageal cancer, PET/CT, adenocarcinoma, staging, SUV, preoperative treatment

## Abstract

Esophageal adenocarcinoma, including adenocarcinoma of the gastroesophageal junction, is uncommon in the United States, but is associated with a rising incidence in young adults, and has a traditionally poor prognosis. Despite the incremental benefits that have been made with multimodality approaches to locally advanced disease, most patients will go on to develop metastatic disease, and long-term outcomes remain suboptimal. Over the last decade, PET-CT has emerged as a key tool in the management of this disease, with several prospective and retrospective studies evaluating its role in this disease. Herein, we review the key data pertaining to the use of PET-CT in the management of locally advanced esophageal and GEJ adenocarcinoma, with a focus on staging, prognostication, PET-CT adapted therapy in the neoadjuvant setting, and surveillance.

## 1. Introduction

Globally, esophageal cancer is the seventh most common cancer and the sixth most common cause of cancer-related death [1]. In 2020, it was estimated that there were approximately 604,000 new cases and 544,000 deaths from esophageal cancer worldwide. The incidence and mortality rates of the two predominant subtypes of esophageal cancer, adenocarcinoma (including adenocarcinoma of the gastroesophageal junction (GEJ)), and squamous cell carcinoma (SCC), differ depending on the geographic location and risk factors of the population. In general, adenocarcinoma has become more common in Western countries in recent years, while SCC is still more common in developing countries. In the United States, adenocarcinoma has surpassed SCC as the most common type of esophageal cancer. According to the American Cancer Society, there will be an estimated 21,560 new cases of esophageal cancer in the US in 2023, with adenocarcinoma being the predominant subtype [2,3]. Despite studies traditionally evaluating both adenocarcinoma and SCC of the esophagus collectively, it is clear that they are fundamentally different in terms of risk factors, histology, genomic characteristics, treatment, and outcomes [4,5]. For this reason, this review will focus exclusively on esophageal adenocarcinoma.

Given the overall poor survival associated with advanced disease, accurate staging at the time of diagnosis is essential in order to avoid potentially toxic therapies and morbid interventions unlikely to improve outcomes. Although randomized trials comparing different staging strategies are lacking, many clinical, endoscopic, and radiological tools have been investigated with varying sensitivities and specificities. Positron emission tomography (PET) combined with computed tomography (CT) imaging utilizes the radiopharmaceutical tracer fluorodeoxyglucose (FDG), a glucose analog labeled with a radioactive material (Fluorine-18), which emits positrons [6]. Positrons are positively charged particles that interact with electrons in the body, producing gamma rays that are detected by the PET scanner and used to create a 3D image of the metabolic activity in the body. The tracer is taken up by cells in the body that are actively using glucose with a high avidity for both esophageal cancer cells and has become a widely utilized imaging modality for the staging of this disease.

Due to its high sensitivity and specificity in the detection of both locoregional and distant metastatic disease, the use of PET-CT has a higher value in locally advanced over early-stage disease. The treatment of locally advanced esophageal cancer has evolved over the last two decades from primary surgical resection to neoadjuvant chemoradiation (chemoRT) and more recently to the addition of adjuvant immunotherapy in those that fail to achieve a pathologic complete response (pCR) [7,8]. For distal and GEJ adenocarcinomas, perioperative chemotherapy is also a standard of care [9]. Improved detection of locoregional lymph node metastases or distant metastatic disease ultimately improves the accuracy of staging, allowing for the most appropriate therapeutic intervention. Similarly, the use of repeat imaging during preoperative therapy allows for restaging and the identification of those that develop metastatic disease, preventing unnecessary surgery.

PET-CT has more recently demonstrated benefits over more conventional imaging, such as CT or magnetic resonance imaging (MRI), in its ability to utilize functional information gained about the disease to predict the response to treatment and long-term outcomes at baseline during and after preoperative treatment. Multiple retrospective reviews suggest that parameters evaluated on baseline PET-CT images may predict those less likely to respond to preoperative therapy and ultimately have a shortened disease-free interval as well as overall survival. The ability to act on this important predictive and prognostication information that can be gained from PET-CT imaging is perhaps its most unique value in the management of patients with esophageal cancer. It has been demonstrated that switching to alternative therapies where there has been an insufficient response to repeat imaging can improve patient outcomes. 

## 2. Staging for Localized Disease

Accurate pre-treatment staging of esophageal and GEJ cancer is an essential component of the appropriate management of patients with this disease. For the purpose of this review, the umbrella term “esophageal cancer” will be used to denote esophageal and GEJ adenocarcinomas. The 8th Edition of the American Joint Committee on Cancer (AJCC) determines the stage of esophageal tumors based on the assessment of the tumor, node, and metastasis (TNM) classification [10]. Traditionally, CT and endoscopic ultrasound (EUS) were the standard modalities employed for the staging of esophageal cancer [11]. However, improved diagnostic accuracy from the routine incorporation of PET-CT imaging is supported by several series and has become a key tool in the staging of locally advanced esophageal cancer [12].

The most significant advantage of PET-CT imaging over conventional CT imaging and EUS is in the detection of distant metastases. The American College of Surgeons Oncology Group Z0060 prospective, multi-institutional study evaluated 189 patients (84% adenocarcinoma) enrolled from 2000 to 2004 with potentially resectable esophageal cancer, staged as T1-3, N0-1, and M0-1a (using the American Joint Committee on Cancer 5th edition staging) based on pre-operative CT, MRI, and bone scans. PET-CT resulted in the identification of biopsy-confirmed M1b disease in 4.8% of patients prior to resection, in addition to suspected distant metastases in a further 9.5%, although not biopsy proven [13]. 

Lowe et al. conducted a prospective study of 75 patients with newly diagnosed esophageal cancer (90% adenocarcinoma), evaluating the staging accuracy of PET-CT, CT, and EUS [14]. PET-CT imaging was reported to have a sensitivity and specificity for the detection of distant metastatic disease of 81% and 91%, respectively, compared with 81% and 82%, respectively, for CT and 73% and 86% for EUS [14]. Furthermore, a meta-analysis including studies that evaluated both adenocarcinoma and SCC demonstrated that PET-CT was associated with a significantly improved diagnostic performance in the diagnosis of distant metastatic disease, reporting a sensitivity and specificity of 71% and 93%, respectively, compared with 52% and 91% for conventional CT [15]. 

The clinical impact of improved upfront identification of distant metastatic disease prior to initiation of treatment was demonstrated by You et al. In a prospective multicenter study of 491 esophageal cancer patients, PET-CT imaging resulted in clinically important changes to treatment in 24% of patients compared with conventional imaging modalities [16]. Specifically, 21.8% of patients had their disease upstaged, while 2.2% were downstaged. Of note, this study did not differentiate between adenocarcinoma and SCC in their analysis. 

While PET/CT scans can better identify metastatic disease, it does not appear to improve the staging of the primary tumor and locoregional lymph nodes. PET-CT imaging has not been shown to be superior to conventional CT in the detection of local nodal involvement and is likely inferior to EUS locoregional staging [17,18]. In cases where locoregional lymph nodes were known to be involved based on pathological or cytological analysis in a predominately adenocarcinoma population (124/148; 84%), EUS was able to detect the malignant lymph nodes in 86% of cases, compared to just 44% for PET-CT. Similarly, PET-CT does not appear to offer any benefit over EUS in the evaluation of the depth of invasion of the primary tumor [14].

## 3. Prognostication

For patients with locally advanced esophageal cancer, the clinical stage and pathologic response to neoadjuvant treatment are typically the most important prognostic factors for long-term disease-free survival (DFS) and overall survival (OS) [19,20,21,22,23]. The prognostic value of FDG uptake on PET-CT imaging was initially reported by Fukunaga et al. in 1998, in which patients noted to have a maximum standardized uptake value (SUV_max_) of >7 on imaging prior to resection had a worse prognosis than those with a SUV_max_ < 7 [24].

Since that time, there have been several efforts to utilize PET-CT characteristics as a tool for predicting disease outcomes. The value of PET-CT in prognostication, however, is dependent on the timing of the imaging, the treatment received, associated changes in PET-CT values, as well as the cut-offs used.

### 3.1. Pretreatment

Given the detailed anatomic staging and ability to detect the distant metastatic disease as described above, baseline PET-CT from the skull base to mid-thigh is recommended prior to the commencement of preoperative therapy in locally advanced disease [25]. Beyond anatomic staging, PET-CT parameters such as the SUV_max_, metabolic tumor volume (MTV), and total lesion glycolysis (TLG) have been investigated as tools to predict patient outcomes. MTV refers to the volume of the metabolically active tumor, while TLG is a product of the mean SUV and MTV that combines volumetric and metabolic information [26].

The prognostic value of SUV_max_ has been widely studied and has been the subject of a meta-analysis [27,28,29,30]. In this meta-analysis of 10 studies with a predominance of adenocarcinoma, all of the studies examined the pretreatment baseline SUV_max_ and its prognostic value for either OS, DFS, or both [27]. The meta-analysis excluded studies where the SUV_max_ was taken from metastatic sites. The combined hazard ratio was 1.86 and 2.52, indicating that a higher primary tumor SUV_max_ was associated with a worse OS and DFS, respectively. 

More recently, Mantziari et al. described the prognostic value of a number of PET-CT parameters on baseline imaging in patients with locally advanced esophageal cancer (53% adenocarcinomas) who underwent surgery [30]. Among 86 patients, 82% of whom received preoperative chemoRT, included for evaluation, a high baseline SUV_max_ on PET-CT was associated with higher clinical T staging (β coefficient 6.61, 95%CI 2.40, 10.81, *p* = 0.002) and node positivity (β coefficient 4.12, 95%CI 0.29, 7.95, *p* = 0.038). A SUV_max_ of >8.25 g/mL was predictive of a ≥cT3 stage with a sensitivity of 84% and a specificity of 68% (AUC = 0.816, 95%CI = 0.704–0.928, *p* < 0.001). A SUV_max_ of >12.7 g/mL was also associated with an earlier tumor recurrence within the first year postoperatively (70.4% sensitivity and 64.4% specificity) and shorter DFS (median 13 vs. 56 months, *p* = 0.030). This study did include patients with both adenocarcinoma and SCC histologies and, when adenocarcinoma tumors were analyzed separately, there was no statistically significant association between DFS and SUV_max_. On the other hand, in another study evaluating a cohort of 45 patients with esophageal adenocarcinoma only, univariate analysis has demonstrated baseline SUV_max_ to be significantly associated with both inferior OS (*p* = 0.008) and DFS (*p* = 0.015) [29]. 

A similar study retrospectively evaluated PET-CT parameters prior to neoadjuvant chemoRT [31]. Within this cohort of 113 patients, 32% had adenocarcinomas. Interestingly within this study, the SUV_max_ and SUV_avg_ in lymph nodes but not the primary tumor was associated with a poor prognosis. This observation may be related to the heterogeneity that exists in the primary tumor with areas of necrosis particularly in larger lesions that could reduce the average and maximum SUV measured. 

Methods to overcome the limitations of SUV and better represent this tumor heterogeneity have been developed with a promising role in prognostication at baseline. Two such PET-CT parameters that have been investigated are MTV and TLG [26]. 

The prognostic value of MTV and TLG derived from pretreatment PET-CT in patients with esophageal cancer has been analyzed in several studies [32,33]. In a large meta-analysis including 1294 patients with both histologies included in the cohort, the hazard ratios for increased functional parameters MTV and TLG for OS were 2.26 and 2.23, respectively [34]. 

Malik et al. reported on a cohort of 150 esophageal cancer patients (75.3% with adenocarcinomas) and found that an MTV of <23.4 cm^3^ (*p* = 0.0001), SUV_max_ <  4.1 (*p* = 0014), EUS T1/2 stage (*p* < 0.0001), EUS N0 stage (*p* < 0.0001), and clinical stage I/II (*p* < 0.0001) were all significantly associated with improved OS, with MTV < 23.4 cm^3^ (*p* = 0.004), EUS T1/2 stage (*p* = 0.01), and EUS N0 stage (*p* = 0.01) significant in multivariate analysis [32].

Prospective evaluation of tumor functional spatial extent such as MTV and TLG on baseline PET-CT imaging also outperformed SUV in the ability to predict response to therapy. In a study of 50 patients with esophageal cancer (28% adenocarcinomas) treated with chemoRT, responses were classified as complete, partial, or nonresponders by RECIST criteria; partial or complete responders were confirmed by biopsy. Baseline pretreatment SUV_max_ was not predictive of response (*p* = 0.29), although those that did have a complete response had a lower SUV_max_ pretreatment (8.1 ± 4.1) than those with a partial response and no response (10.2 ± 3.7 and 10.2 ± 3.9, respectively). Pretreatment MTV and TLG did, however, allow for significant differentiation of the three response groups; with values for both parameters at baseline associated with a higher likelihood of response to subsequent neoadjuvant chemoRT (*p* ≤ 0.002) [35]. In particular, TLG was the parameter that allowed for the best differentiation between the three response groups (74 ± 75, 179 ± 143, and 385 ± 226 g for CR, PR, and NR patients, respectively; *p* < 0.0010).

### 3.2. Metabolic Response to Treatment

While SUV, MTV, and TLG have been demonstrated to be associated with clinical outcomes, baseline assessment alone does have limitations as it does not account for any response or resistance to therapy. As such, studies have focused on both the absolute SUV_max_ uptake value following neoadjuvant treatment imaging as well as the dynamics of change from baseline. The percentage change in SUV_max_ has been demonstrated to be superior to EUS and standard CT imaging in predicting the presence of residual disease at the time of surgery [36,37,38]. Based on this, reassessment with repeat PET-CT prior to surgery with the evaluation of the change in parameters has been of significant interest as a more sophisticated tool for prognostication.

In a retrospective evaluation of 103 patients with esophageal cancer (87% adenocarcinomas) treated with preoperative chemoRT at MD Anderson Cancer Center, patients were evaluated before or after chemoRT with EUS, CT of the chest/abdomen, and PET-CT imaging [39]. From the post-chemoRT evaluations, the investigators employed thresholds for each modality to differentiate pathologic responders from nonresponders. The thresholds were used to assess the sensitivity, specificity, and accuracy of each modality in predicting pathologic nonresponse (defined as ≥10% viable cancer in the primary tumor). Post-neoadjuvant chemoRT SUV_max_ ≥ 4 of the primary tumor was the most specific and accurate (specificity 84%, accuracy 76%) modality in the prediction of pathologic nonresponse. The same threshold was also utilized to predict long-term outcomes. Only post-chemoRT SUV_max_ ≥ 4 predicted long-term survival, with the 18-month OS rate of patients with a post-chemoRT SUV_max_ ≥ 4 being 34% compared with 77% for patients with an SUV < 4 (*p* = 0.01). Univariate analyses demonstrated that post-chemoRT SUV_max_ was the only radiological parameter that predicted long-term survival, while multivariate analysis of the three modalities showed that only post-chemoRT PET-CT and CT thickness were independent predictors of long-term survival. 

In another contemporaneous report from this same group, the accuracy of the post-chemoRT PET-CT in detecting residual disease was assessed [40]. An abnormal PET-CT scan had an accuracy of 69% in predicting residual malignancy. However, the false-negative rate for identifying residual malignancy was 18% and there was not a SUV_max_ cutoff that could identify with absolute accuracy patients who had achieved a pCR to pre-operative chemoRT. As such, the authors concluded that esophagectomy should be considered even if the post-chemoRT PET-CT scan is normal.

The changes in MTV and TLG have also been investigated as more sophisticated prognostic tools in those undergoing chemoRT. Roedl et al. demonstrated in 51 patients with locally advanced esophageal adenocarcinoma undergoing chemoRT that with a threshold of a 63% decrease in MTV, the PET–CT volume is able to predict a histopathologic response (defined as <10% viable cells in the surgical specimen) with a sensitivity of 91% and a specificity of 90% (odds ratio 4.9, 95% CI 2.2–11.1, *p* < 0.001) [41]. The highest accuracy was observed using TLG, whereby a decrease of >78% predicted the pathologic response with a sensitivity and specificity of 91% and 93%, respectively (odds ratio 12.1, 95% CI 3.3–45.2, *p* < 0.001). Metabolic responders, defined as >63% decrease in MTV between the baseline and post-neoadjuvant chemoRT PET-CT imaging had a longer mean time to recurrence compared to nonresponders (29.4 months (95% CI 26.3–32.6) vs. 16 months (95% CI 13.7–18.3), *χ*^2^: 25.5, *p* < 0.001). OS was also significantly improved in metabolic responders compared to nonresponders (34.1 months (95%CI: 31.4–36.8) vs. 21.8 months (95% CI: 19.4–24.2), *χ*^2^: 14.9, *p* < 0.001).

### 3.3. Early Response Assessment

A lack of pathological response to neoadjuvant therapy has also clearly been demonstrated as a poor prognostic sign and may even be worse than that of primary surgery alone [42]. Similarly, for the PET-CT parameters discussed above, a lack of response to neoadjuvant therapy and persistent increased SUV_max_ heralds a worse overall outcome. Ineffective neoadjuvant chemoRT or chemotherapy may cause unnecessary adverse events and allow time for tumor progression on treatment. Therefore, the ability to predict the response to neoadjuvant treatment, and more importantly identify those not responding, would provide critical information for decision making.

Given the ability to reliably predict the response to treatment and long-term outcomes with the change in PET-CT SUV_max_ post neoadjuvant treatment, efforts have been made by German investigators to identify responders early into preoperative chemotherapy with repeat imaging after just 14 days of treatment [43,44]. PET-CT imaging was performed at baseline and after 2 cycles of 5-fluourouracil/cisplatin as part of two prospective phase II studies including 40 patients with locally advanced GEJ adenocarcinoma [43]. Following one cycle of chemotherapy, a repeat PET/CT scan on Day 14 demonstrated that the decrease in FDG uptake was significantly higher in clinical responders vs. nonresponders (responders: −54% ± 17%, median: −54%; nonresponders: −15% ± 21%, median: 15%; *p* < 0.001) For this analysis, response was defined as at least 50% reduction in the size of the primary tumor, as measured by endoscopy and imaging studies. A reduction in FDG uptake of ≥35% was found to have the greatest accuracy in differentiating between responders and nonresponders. By applying the 35% cutoff value, the sensitivity and specificity were 93% (CI, 68% to 100%) and 95% (CI, 77% to 100%), respectively, for identifying responses. Positive and negative predictive values for a clinical response were 93% (CI, 68% to 100%) and 95% (CI, 77% to 100%), respectively. Similarly, this cutoff of a metabolic response to treatment provided high sensitivity and specificity for the prediction of a pathological response (defined as no or only a few scattered residual cells; Mandard tumor regression grades 1 and 2) of 89% and 75%, respectively. In patients with a metabolic response, median progression-free survival (PFS) was 16 months with a 2-year OS rate of 49%, whereas PFS was substantially shorter at 9 months (*p* = 0.01) with a 2-year OS rate of just 9% for patients without a metabolic response (*p* = 0.04).

Prospective validation of these findings was then performed by Ott and colleagues [44]. In this study, 65 patients with locally advanced esophageal adenocarcinoma were evaluated with PET-CT at baseline and again 14 days after 1 cycle of chemotherapy. Fifty-six patients were evaluable as metabolic responders or not. Disease progression following treatment was noted in six patients, five of whom were nonresponders. Of the 50 that underwent surgery, the presence of a metabolic response was correlated with a pathological response, with a pathological response rate of 44% in metabolic responders, compared to just 5% in those without a metabolic response (*p* = 0.001). This cutoff of 35% was also prospectively validated as a prognostic tool for long-term outcomes. The median OS reported was just 18 months for nonresponders whereas, after a median follow-up time of 42 months, it was not reached in the responders group (*p* = 0.01). Other retrospective series have supported the prognostic value of early metabolic response to induction chemotherapy [45].

All of this evidence led to the hypothesis that if a poor response could be accurately predicted on PET-CT imaging, early modification of treatment may salvage some patients and improve outcomes. 

## 4. Neoadjuvant Treatment Modification Based on PET-CT

Building on their identification of a prognostic SUV_max_ cut-off after one cycle of chemotherapy, the German MUNICON trial used the results of PET imaging to guide therapy in patients with locally advanced GEJ adenocarcinoma [46]. Patients received one cycle of 5-FU/cisplatin chemotherapy, followed by PET-CT assessment for metabolic response (pre-defined as a tumor glucose SUV of ≥35% based on their prior work above) two weeks later. Responders continued neoadjuvant chemotherapy for a total of twelve weeks prior to resection, while nonresponders proceeded directly to resection. The rationale for stopping treatment in PET-CT nonresponders was to avoid chemotherapy-associated toxicity in patients not deriving benefits. Of the 110 patients who were evaluable, 49% had a response on PET-CT imaging. At a median follow-up of 2.3 years, metabolic nonresponders demonstrated a median event-free survival (EFS) of 14.1 months, and a median OS of 25.8 months. In metabolic responders, a median EFS of 29.7 months was observed, and median OS was not reached. Further, major histological remissions (defined as <10% residual tumor) were noted in 58% of metabolic responders, and no histological response was noted in metabolic nonresponders. Compared with prior prospective data of metabolic nonresponders who completed 3 months of pre-operative chemotherapy and then proceeded to surgery and had a median OS of 18 months [44], outcomes from patients in the MUNICON trial suggested that PET non-responders were not disadvantaged by early discontinuation of ineffective chemotherapy, and proceeding directly to surgery [44].

If the MUNICON study demonstrated that stopping ineffective chemotherapy did not comprise outcomes following surgery, the follow-up question is whether PET-CT nonresponders can be salvaged with an alternative regimen or treatment (Table 1). The MUNICON II trial aimed to answer this question [47]. In this study, 56 patients with locally advanced GEJ adenocarcinomas underwent PET-CT imaging after 2 weeks of induction chemotherapy with 5 FU/cisplatin, following which responders continued chemotherapy for 3 months. PET-CT nonresponders proceeded to salvage neoadjuvant chemoRT with cisplatin, followed by resection. The primary objective to increase the R0 resection rate in PET-CT nonresponders from 74% to 94% was not met. PET-CT nonresponders had inferior 1-year PFS (57% ± 10% versus 74% ± 8%, *p* = 0.035) and a trend toward inferior 2-year OS (42% ± 11% versus 71 ± 8%; HR 1.9; 95% CI, 0.87–4.24; *p* = 0.10). However, these results do not definitively prove that such an approach is ineffective. In this study, the administered radiation dose of 32 Gy was relatively low, and cisplatin alone was administered concurrently with RT in patients who already had a poor metabolic response to 5-FU/cisplatin.

The observation that changing the chemotherapy regimen in nonresponders offers benefit was first made by Ilson et al. from our group in a phase II study of 55 patients with resectable tumors of the esophagus or GEJ, including patients with adenocarcinoma (75%), squamous cell carcinoma (22%), and poorly differentiated carcinoma (35%) [48]. Patients received induction cisplatin/ irinotecan, followed by chemoRT with cisplatin/irinotecan and surgery. In a retrospective analysis, changes in PET-CT with a SUV_max_ ≥ 35% following induction chemotherapy strongly predicted improved outcomes compared with those with a SUV_max_ decrease of <35%. These outcomes included the pCR rate (32% versus 4%, *p* = 0.009), R0 resection rate (84% versus 57%, *p* = 0.02), median PFS (24.1% versus 7.7%, *p* = 0.02), and a non-significant trend toward improved median OS (40.2 months versus 25.5 months, *p* = 0.29). Of particular note, in four patients who had frank progression on PET-CT after induction chemotherapy, three achieved durable complete responses upon switching to alternative chemotherapy regimens during radiotherapy (RT).

This observation was subsequently confirmed on a retrospective review of 201 patients with esophageal/GEJ adenocarcinomas, who underwent induction chemotherapy prior to chemoRT for locally advanced disease [49]. Of the 88 PET-CT nonresponders following chemotherapy, 38 (43%) were switched to an alternative chemotherapy during RT. The pCR rate for PET-CT nonresponders who changed therapy was 10% versus 3% for those who did change chemotherapy (3%), which was not statistically significant (*p* = 0.32). The median PFS for PET-CT nonresponders who changed chemotherapy was superior to those who did not change chemotherapy (17.9 months versus 10 months, *p* = 0.01) although the median OS was similar, 25.8 months versus 25.3 months, *p* = 0.18). Five-year OS rates were 48% vs. 25% for PET non-responders who did and did not change chemotherapy during RT, respectively (*p* = 0.18).

Building on the initial anecdotal observations by Ilson and colleagues, the Cancer and Leukemia Group 80803 study employed the strategy of alternating chemotherapy in PET-CT nonresponders [50]. Two hundred and forty-one patients with locally advanced esophageal and GEJ adenocarcinoma were enrolled, all undergoing PET-CT imaging at baseline, followed by induction chemotherapy with randomization to either carboplatin/paclitaxel or FOLFOX. PET-CT responders continued on the same chemotherapy treatment concurrently with RT, while PET-CT nonresponders switched to the alternative regimen with RT. The primary endpoint of increasing the pCR rate from a historical control of 5% (generated from the MSKCC data) in the PET-CT nonresponders who changed chemotherapy during RT. The study met its primary endpoint: the pCR rate was 18% in FOLFOX nonresponders who switched to carboplatin/paclitaxel and 20% in the carboplatin/paclitaxel nonresponders who switched to FOLFOX. At a median follow-up of 5.2 years, the median OS was 48.8 months (95% CI, 33.2 to not estimable) for metabolic responders, and 27.4 months (95% CI, 19.4 to not estimable) for nonresponders, which did not meet statistical significance (*p* = 0.107). Of note, the pCR rate among PET-CT responders to induction carboplatin/paclitaxel was 14.1% (95% CI, 6.6 to 25.0) compared to 40.3% (95% CI, 28.9 to 52.5) in metabolic responders to induction FOLFOX, and this also correlated with improved OS for the induction FOLFOX responders group (5-year OS 53% versus 43.9%) compared with the induction carboplatin/paclitaxel responders group. As such, this study suggested that a PET-directed approach with induction FOLFOX might be optimal.

A major limitation of the CALGB study is that there was no control group that did not evaluate a PET-CT-directed strategy. As such, our group retrospectively compared a PET-CT-directed approach vs. standard chemoRT [51]. We analyzed 451 patients who underwent PET-directed chemoRT with either induction FOLFOX (*n* = 70), carboplatin/paclitaxel (239), or chemoRT with carboplatin/paclitaxel without induction therapy (*n* = 142). Patients who received induction FOLFOX had significantly higher rates of both pCR (33% versus 16%, *p* = 0.004) and near-pCR (defined as ypN0 and ≥90% treatment response) (57% versus 33%, *p* < 0.001), compared with those who received induction carboplatin/paclitaxel. The rates of pCR (33% versus 22%, *p* = 0.10) and near-pCR (57% versus 42%, *p* = 0.04) were also greater in those who received induction FOLFOX compared with those who received standard carboplatin/paclitaxel and RT, although only statistically significant for near-pCR. Furthermore, the 2-year DFS was higher in the induction FOLFOX group compared with both the induction carboplatin/paclitaxel group (68% versus 50%, *p* = 0.01), and the standard carboplatin/paclitaxel and RT group (68% versus 44%, *p* < 0.001). Based on this analysis and the CALGB 80803 results, our institutional practice is to treat patients with a PET-adjudicated approach utilizing induction FOLFOX.

**Table 1:** Key completed trials of ^18^FDG-PET-CT-directed management in locally advanced esophageal/GEJ adenocarcinoma.

Trial	Phase	Patient Population	N	Definition of PET Response	Treatment	Primary Endpoint	Other Key Findings
MUNICON [46]	2	Locally advanced GEJ adenocarcinoma	119	≥35% reduction in SUV_max_	All patients: induction platinum and 5-FU × 2 weeks Responders: continued platinum and 5-FU × 12 weeks, then resection Non-responders: direct to resection	mOS Responders: NR Non-responders: 25.8 months	mEFS Responders: 29.7 mos; non-responders: 14.1 mos Major histological response Responders: 58% Non-responders: 0%
MUNICON II [47]	2	Locally advanced GEJ adenocarcinoma	56	≥35% reduction in SUV_max_	All patients: induction platinum and 5-FU × 2 weeks Responders: continued platinum and 5-FU × 12 weeks then resection Non-responders: radiation (32 Gy) and cisplatin, then resection	R0 resection rate Responders: 82% Non-responders: 70% (*p* = 0.51)	mOS Responders: NR Non-responders: 18.3 months Time to progression Responders: NR Non-responders: 14.4 months
Ilson et al., 2011 [48]	2	Resectable SCC or adenocarcinoma of the esophagus or GEJ	55	≥35% reduction in SUV_max_	All patients: induction cisplatin and irinotecan weeks 1 to 5, then chemoradiotherapy weeks 7 to 11	pCR rate Overall: 16% PET Responders: 32% PET Non-responders: 4% (*p* = 0.009)	R0 resection Responders: 84% Non-responders: 57% (*p* = 0.02) mPFS Responders: 24.1 mos; non-responders: 7.7 mos (*p* = 0.02) mOS Responders: 40.2 mos Non-responders: 25.5 mos (*p* = 0.29)
CALGB 80803 [50]	2	Locally advanced esophageal or GEJ adenocarcinoma	241	≥35% reduction in SUV_max_	Patients randomized 1:1 to CP or FOLFOX. Responders: continued same chemotherapy with radiation Non-responders: switched to the alternative chemotherapy regimen with radiation	pCR Rate in PET Non-responders after switching chemotherapy FOLFOX → CP: 18% CP → FOLFOX: 20%	pCR rate in Responders FOLFOX: 40.3% CP: 14.1% mOS Responders: 48.8 mos: Non-responders: 27.4 mos (*p* = 0.107)
AGITG DOCTOR [52]	2	Resectable esophageal adenocarcinoma	124	≥35% reduction in SUV_max_	All patients: induction cisplatin and 5-FU × 1 cycle Responders: continued received a 2nd cycle, then resection Non-responders: randomized to DCF × 2 cycles then resection, or DCFRT then resection	Major histological response * Responders: 7% Non-responders DCF: 20% Non-responders DCFRT: 63%	PFS at 36 months Responders: 47% Non-responders DCF: 29% Non-responders DCFRT: 46% OS at 60 months Responders: 53% Non-responders DCF: 31% Non-responders DCFRT: 46%

* Major histological response defined as <10% residual tumor. Abbreviations: GEJ: gastroesophageal junction, SCC: squamous cell carcinoma, N: patient number, SUV: maximum standardized uptake values, 5-FU: 5-Fluorouracil, DCF: Docetaxel + Cisplatin + Fluorouracil, DCFRT: DCF + Radiotherapy, mOS: median Overall Survival, mos: months, pCR: pathologic complete response, mEFS: median Event-Free Survival, mPFS: median Progression-Free Survival, NR: Not Reached.

The AGITG DOCTOR trial further demonstrates that early metabolic response adjudicated by sequential PET-CT imaging plays an important role in the management of patients with esophageal adenocarcinoma and supports tailoring of treatment based on early metabolic response assessment by PET-CT on day 15 [52]. Patients who did not have a metabolic response on PET-CT after induction 5-FU/cisplatin were randomized in a 1:1 fashion to receive two cycles of 5-FU/cisplatin with docetaxel (DCF; Arm A) or DCF + 45 Gy RT (Arm B), followed by esophagectomy; PET responders received a second cycle of 5-FU/cisplatin. The primary endpoint was major histological response (<10% residual tumor), while secondary endpoints were OS, PFS, and locoregional recurrence. The primary tumor analysis was carried out in 123 patients, including 45 responders, 30 in Arm A, 35 in Arm B, and 13 others (who had no Day 15 PET-CT). Major histological response was seen in 7% (95% CI, 2 to 18) of the early metabolic responders group, and among metabolic nonresponders, major histological response was seen in 20% (95% CI, 10 to 37) of patients in Arm A and 63% of patients in Arm B (95% CI, 46 to 77), median PFS was 27 months (95% CI, 12 to not reached) in patients with early metabolic responses, 22 months (95% CI, 14 to 27) for Arm A, and 22 months (95% CI, 14 to not reached) for Arm B. A longer median OS of 61 months (95% CI, 21 to not reached) was observed in those with early metabolic responses, compared with those in Arm A and Arm B (30 months (95% CI. 17 to 55), and 35 months (95% CI, 20 to not reached), respectively. However, the difference in 5-year OS rates appeared less pronounced; 53% (95% CI, 37 to 67) for early metabolic responders versus 31% (95% 16 to 48) for Arm A and 46% (95% CI, 29 to 61) for Arm B. These data continue to support the aforementioned studies in that early metabolic response to induction chemotherapy is associated with favorable OS, PFS, and low local recurrence rates. With respect to nonresponders, while the findings from the AGITG DOCTOR trial require validation in a larger study, they suggest that tailoring of treatment based on early PET-CT non-response, for example, with the addition of RT and altering chemotherapy, may help to close the gap between early metabolic responders and nonresponders and result in similar long-term outcomes between the two groups.

## 5. Surveillance

Despite improvements in perioperative systemic and locoregional therapies, disease recurrence rates remain high with close surveillance post-definitive therapy suggested. With the majority of recurrences occurring in the first 2 years following definitive therapy, imaging is recommended during this time [25,53]. The current recommendation from the National Comprehensive Cancer Network (NCCN) is for CT chest abdomen and pelvis with contrast to be performed every 6 months for up to 2 years [25]. Several retrospective series have demonstrated that this routine surveillance post-curative therapy will likely detect recurrence earlier than that of symptom-prompted imaging strategies [54,55,56]. Given the evidence for the detection of occult metastases at the time of diagnosis with PET-CT over standard CT, it is conceivable that PET-CT may be even more effective at detecting recurrence post-treatment. A meta-analysis by Goense et al. reported that in patients with esophageal cancer following treatment with curative intent, PET-CT can detect recurrence with a pooled sensitivity and specificity of 96% and 78%, respectively [57]. However, recurrent esophageal cancer remains incurable with current therapy and there is no standard role for metastasectomy. As such, it is likely that any potential ability of PET-CT imaging to detect recurrent/metastatic disease earlier than conventional contrast-enhanced CT imaging will not translate into a significant OS benefit based on currently available therapies. As such, PET-CT is not currently recommended for surveillance following definitive treatment of this disease. 

## 6. Conclusions

We describe herein the clinical utility of PET-CT as an important imaging modality in the management of locally advanced gastroesophageal adenocarcinoma. The role of PET-CT in improving the diagnostic accuracy for staging, in addition to its use as a predictive and prognostic tool, is supported by several prospective and retrospective series. Furthermore, the data supporting the use of PET-CT-adapted therapy through switching chemotherapy during radiation in nonresponders to induction chemotherapy, are compelling and incrementally improve outcomes for this group with a traditionally poor prognosis. As treatment evolves with more modern perioperative chemotherapy regimens and the addition of immunotherapy, further prospective studies will be required to validate the use of PET-CT to predict outcomes. Future applications of PET-CT may include early identification of patients most likely to achieve unfavorable outcomes with standard therapy and the incorporation of novel and experimental therapeutic strategies early in their treatment paradigm.

## Data Availability

Not applicable.
